# A large-scale CRISPR screen and identification of essential genes in cellular senescence bypass

**DOI:** 10.18632/aging.102034

**Published:** 2019-06-20

**Authors:** Xuehui Liu, Lei Wei, Qiongye Dong, Liyang Liu, Michael Q. Zhang, Zhen Xie, Xiaowo Wang

**Affiliations:** 1 MOE Key Laboratory of Bioinformatics; Bioinformatics Division and Center for Synthetic and Systems Biology, Beijing National Research Center for Information Science and Technology, Department of Automation, Tsinghua University, Beijing 100084, China; 2 Department of Basic Medical Sciences, School of Medicine, Tsinghua University, Beijing 100084, China; 3 Department of Biological Sciences, Center for Systems Biology, The University of Texas, Richardson, TX 75080, USA; 4 Present address: State Key Laboratory of Medical Molecular Biology, Department of Pathophysiology, Institute of Basic Medical Sciences, Chinese Academy of Medical Sciences and Peking Union Medical College, Beijing 100730, China; 5 Present address: Key Laboratory of Intelligent Information Processing, Advanced Computer Research Center, Institute of Computing Technology, Chinese Academy of Sciences, Beijing 100190, China

**Keywords:** aging, cellular senescence, bypass, CRISPR, SASP

## Abstract

Cellular senescence is an important mechanism of autonomous tumor suppression, while its consequence such as the senescence-associated secretory phenotype (SASP) may drive tumorigenesis and age-related diseases. Therefore, controlling the cell fate optimally when encountering senescence stress is helpful for anti-cancer or anti-aging treatments. To identify genes essential for senescence establishment or maintenance, we carried out a CRISPR-based screen with a deliberately designed single-guide RNA (sgRNA) library. The library comprised of about 12,000 kinds of sgRNAs targeting 1378 senescence-associated genes selected by integrating the information of literature mining, protein-protein interaction network, and differential gene expression. We successfully detected a dozen gene deficiencies potentially causing senescence bypass, and their phenotypes were further validated with a high true positive rate. RNA-seq analysis showed distinct transcriptome patterns of these bypass cells. Interestingly, in the bypass cells, the expression of SASP genes was maintained or elevated with *CHEK2*, *HAS1*, or *MDK* deficiency; but neutralized with *MTOR*, *CRISPLD2*, or *MORF4L1* deficiency. Pathways of some age-related neurodegenerative disorders were also downregulated with *MTOR*, *CRISPLD2*, or *MORF4L1* deficiency. The results demonstrated that disturbing these genes could lead to distinct cell fates as a consequence of senescence bypass, suggesting that they may play essential roles in cellular senescence**.**

## INTRODUCTION

Cellular senescence is a cell fate with stable cell cycle arrest triggered by a variety of stimuli, such as telomere attrition, DNA damage, oxidative stress, and oncogene activation [1]. Cellular senescence acts as an important tumor-suppression mechanism, but in the meanwhile, its dark sides may lead to inflammation, tumor promotion, or aging [2]. Senescent cells often exhibit flattened and enlarged morphology, DNA-damage foci and senescence-associated heterochromatin foci (SAHF), as well as altered gene expressions such as the increment of p16^INK4a^, positive staining for senescence-associated β-galactosidase (SA-β­gal) and senescence-associated secretory phenotype (SASP).

It has been a great challenge to understand the regulatory mechanisms in cellular senescence to control cell fate with its benefits while refraining from its side effects. Senescence processes are well known to be mainly controlled by p53–p21 and/or p16–pRB pathways [3, 4]. Disruptions of genes in these pathways may result in escaping the fate of stable cell cycle arrest when facing senescence-inducing stimuli, or in other words, acquiring the ability to bypass senescence [5, 6]. Identifying genes deficiency of which causes senescence bypass (“senescence bypass genes” for short) helps us gain knowledge about the regulatory process of cellular senescence and tumorigenesis, and thus provides potential targets to control cell fates when encountering senescence stimulus for anti-cancer or anti-aging therapy. A number of senescence bypass genes have been unveiled by functional screen methods such as retroviral cDNA libraries [7, 8] and shRNA libraries [9, 10]. These screens usually required isolations and expansions of bypass colonies to amplify the moderate proliferative signatures from a large number of genes in the screen library.

Recent progress on clustered regularly interspaced short palindrome repeats (CRISPR)-associated nuclease Cas9 system provided novel tools for pooled large-scale screens of gene function [11, 12]. In these screens, cells are infected with pooled lentiviral single-guide RNA (sgRNA) library, and the abundance of cells carrying functional sgRNAs, which can be detected by deep sequencing, may be amplified or diminished due to the alterations of cell viability [11, 12], growth rate [13], ability to metastasize [14], or expression of particular biomarkers [15]. Pooled screens by CRISPR-Cas9 knockout libraries show to be more robust, effective and specific compared with traditional screening methods [11, 16].

In this study, we presented a large-scale pooled CRISPR-Cas9 knockout screen in human primary dermal fibroblasts (BJ) to comprehensively identify genes that are essential for the establishment or the maintenance of senescence. We designed and constructed a CRISPR-Cas9 knockout library targeting genes associated with cellular senescence by integrating the information of literature mining, protein-protein interaction (PPI) network, and differential gene expression data from dozens of microarrays, and performed a pooled screen based on the library. We not only confirmed several known senescence bypass genes but also uncovered a dozen novel gene deficiencies that led to senescence bypass. Further validations of these genes showed a high true positive rate of the screen. RNA-seq analysis of these bypass cells exhibited distinct transcriptome patterns. Bypass cells with *MTOR*, *CRISPLD2*, or *MORF4L1* deficiency intriguingly showed a neutralized SASP expression profiles in comparison with senescent cells, and pathways of some age-related neurodegenerative disorders were downregulated in these cells. The results showed that there are distinct consequences of senescence bypass, and implied different roles of these senescence bypass genes in the process of cellular senescence.

## RESULTS

### A pooled CRISPR-Cas9 knockout screen with a well-designed senescence-associated sgRNA library in human fibroblasts for cellular senescence bypass

**Figure 1:**
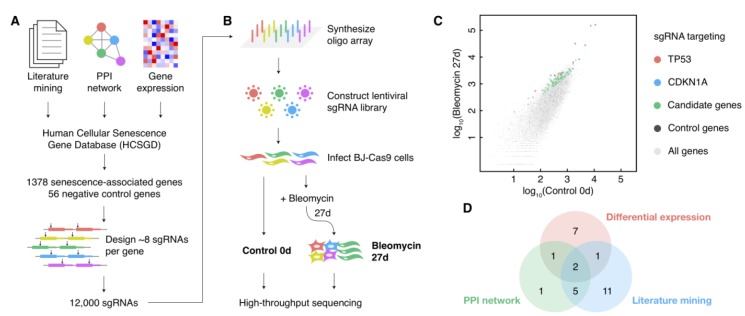
**A large-scale CRISPR knockout screen for cellular senescence bypass.** (**A**) Design of the senescence-associated sgRNA library. (**B**) Schematic diagram of the sgRNA library construction and the CRISPR knockout screen. (**C**) Scatterplot of the normalized reads count of all sgRNAs in control and bleomycin-induced samples. sgRNAs targeting positive control genes (*TP53* and *CDKN1A*) and negative control genes were shown in different colors. (**D**) The source of the candidate senescence bypass genes in the design approach of the senescence-associated sgRNA library.

The pooled screen for cellular senescence bypass relies on the alterations of the cell growth rate in bypass cells, which contains more considerable noise compared with screens by cell viability. Therefore, the coverage of cells infected with a certain sgRNA should be increased to detect the moderate alterations of bypass cell abundances precisely. However, it is hard to passage mortal fibroblasts to a vast amount in comparison with cancer cell lines. Besides, cultivating too many cells during a long time from induction to deep senescence (about one month) is costly and labile. Thus, we designed a senescence-associated sgRNA library to enlarge the coverage and to improve the screen performance consequently (Figure 1A). We selected 1378 genes related to cellular senescence from our Human Cellular Senescence Gene Database (HCSGD) [17] by integrating information of three methods, including literature mining, PPI network and differential gene expression data from dozens of microarrays. In addition, 56 negative control genes were also included. A total number of 12,000 sgRNAs were designed using our CRISPR-ERA platform [18] to target these genes (about eight sgRNAs per gene) and were constructed into a pooled lentiviral library (Figure 1A).

Human fibroblast BJ cells expressed low levels of p16, and thus to be more susceptible to single-gene perturbations to escape from cellular senescence compared with cells with high levels of p16 [19]. Therefore, we performed a CRISPR-Cas9-based single-gene knockout screen on BJ cells to enlarge the efficiency of bypass gene identifications. BJ cells stably expressing Cas9 were infected with the sgRNA lentiviral library. We deeply sequenced library sgRNAs amplified from cell genomes before and 27 days after senescence induction by bleomycin, each with two biological replicates (Figure 1B). The replicates showed a high correlation (Supplementary Figure 1A), and a method based on MA-plot was performed to detect sgRNAs with significantly increased counts in senescent cells compared with pre-induction cells (Figure 1C and Supplementary Figure 1B). Thirty genes with three or more sgRNAs significantly increased (*p*-value < 0.05) in both replicates were selected as candidate senescence bypass genes (Supplementary Table 1). All three methods used to design senescence-associated gene pools each provided a portion of these candidate senescence bypass genes (Figure 1D), indicating the necessity and complementarity of these methods. The screen also identified several previously reported genes such as *TP53*, *CDKN1A*, *ARID3A*, *ITSN2*, *AKT1*, *ATM*, and *ZBTB7A* (Supplementary Table 1).

We also performed a parallel pooled bypass screen induced by oncogenic protein RAS^V12^ to evaluate these candidate bypass genes. BJ cells carrying the same sgRNA lentiviral library were infected with RAS^V12^ lentivirus; 13 days later, the library sgRNAs were amplified from cell genomes, deep sequenced and analyzed by the same approach as the screen induced by bleomycin. As a result, four genes (*TP53*, *CDKN1A*, *AHNAK2*, and *IKBKB*) emerged in both the bleomycin-induced and the RAS^V12^-induced screen. This result suggested that senescence caused by different induction methods may not only share some general but also employ distinct genes or pathways [20].

### Functional characterizations of the candidate senescence bypass genes

**Figure 2:**
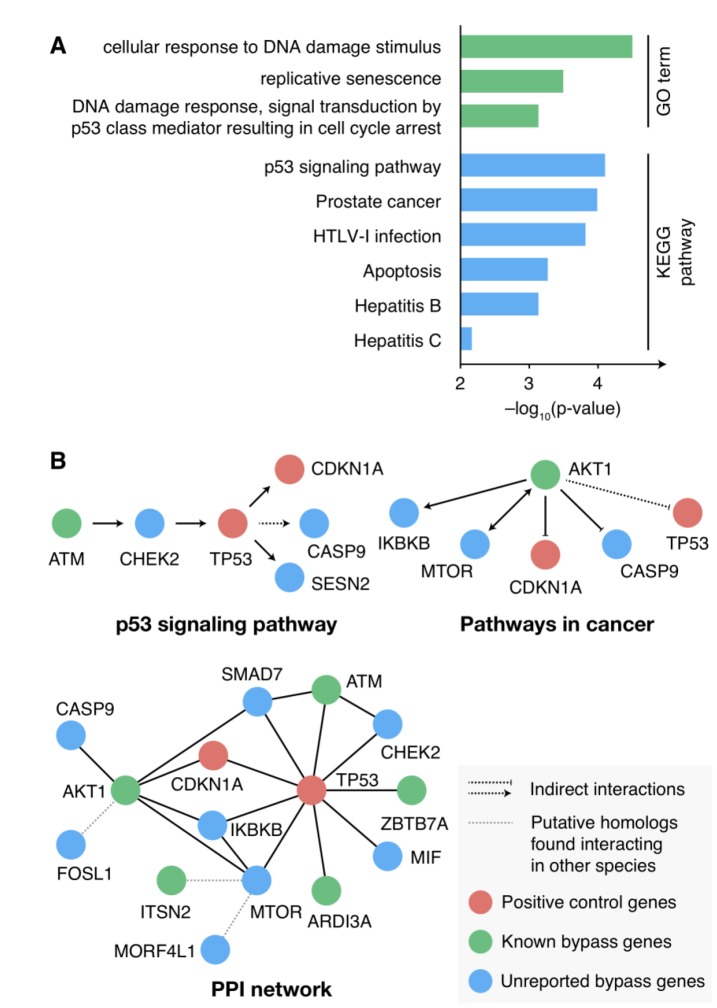
**Function characterizations of the candidate senescence bypass genes.** (**A**) KEGG pathway and GO enrichment results of the candidate senescence bypass genes. The *p*-value was adjusted using the Benjamini-Hochberg procedure. Terms with adjusted *p*-value < 0.01 were shown. (**B**) The candidate genes in senescence-associated pathways and PPI networks.

We first examined the expression profile of the candidate senescence bypass genes in the process of cellular senescence with a set of time-series gene expression data GSE41714 during replicative senescence in human diploid fibroblasts [21]. Among the 30 candidate bypass genes, six showed up-regulated, while three showed down-regulated at the transcriptional level in the process of cellular senescence (Supplementary Figure 2). Next, enrichment analysis of KEGG pathway and gene ontology (GO) was performed to explore the function of the candidate bypass genes. These genes were highly enriched in senescence-associated GO terms and pathways, such as DNA damage response, replicative senescence, p53 signaling, and cancer (Figure 2A). Further investigations of the pathways and PPI network showed *TP53* and *AKT1* as regulatory hubs among the candidate senescence bypass genes (Figure 2B), both of which play critical roles in senescence and tumorigenesis.

**Figure 3:**
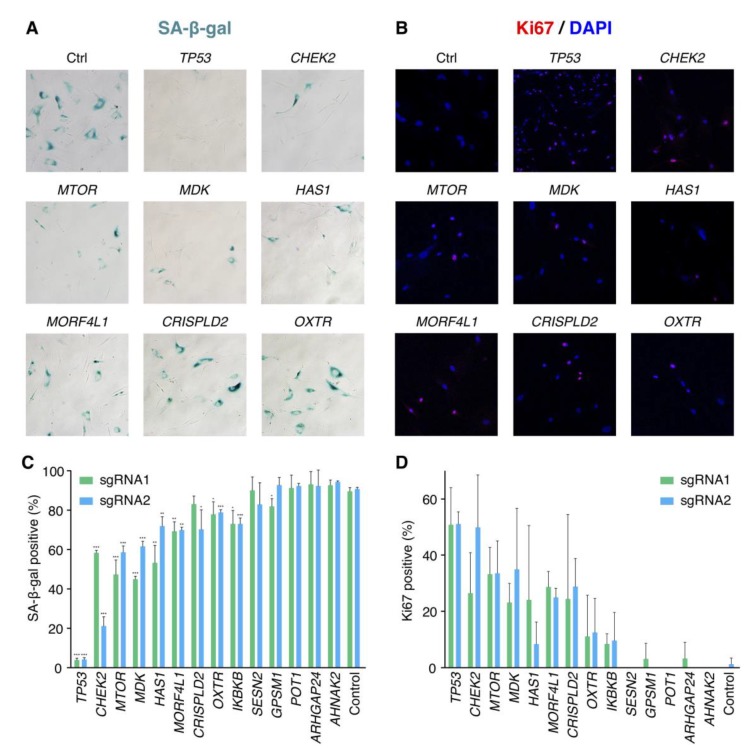
**Validation results of the candidate senescence bypass genes.** (**A**) The senescence bypass caused by different candidate genes knockout was detected with SA-β-gal staining. Representative images were shown. (**B**) Representative images of senescence bypass cells stained by the proliferative marker Ki67. (**C**) Percentage of β-gal positive cells at 27 days after bleomycin induction (n = 3, mean ± SD). *p < 0.05; **p < 0.01; ***p < 0.001 by *t*-test in comparison with control cells. (**D**) Percentage of Ki67 positive cells at 27 days after bleomycin induction in samples knocking-out candidate genes (n = 3, mean ± SD).

We further validated 13 candidate genes without previous knowledge on senescence bypass respectively. For each gene, two significantly increased sgRNAs in the pooled screen were selected for validation. 27 days after bleomycin induction, the extents of senescence bypass were detected with the SA-β-gal assay, the colony formation assay, and the Ki67 immunofluorescence assay (Figure 3A, 3B). Any sample with a significantly reduced ratio of positive SA-β-gal in comparison with senescent cells and more than 10% positive ratio of the proliferative marker Ki67 was identified as a faithful senescence bypass. As a result, 7 of the 13 genes showed senescence bypass with at least one successfully validated sgRNA (Figure 3C–3D). The failure of other validated genes to bypass senescence might be caused by the differences of the microenvironments between the pooled screen and isolated validations or implied that deficiencies of these genes might delay but not bypass cellular senescence.

Some of the successfully validated genes such as *CHEK2*, *MTOR*, and *MORF4L1* were reported previously to be associated with cellular senescence. *CHEK2* is a cell cycle checkpoint regulator and a stabilizer of p53 in the cellular senescence pathway, and *CHEK2* deficiency was shown to disturb cell cycle arrest and confer a predisposition to multiple tumors [22, 23]. *MTOR* also involves the senescence pathway that regulates cell proliferation. Inhibition of *MTOR* by rapamycin showed a suppressed pro-tumorigenic SASP, and a small proportion (< 1%) of BJ cells bypassed senescence at 5 Gy X-irradiation when treated by rapamycin [24]. Moreover, both rapamycin and disruption of mTOR extended the lifespan of mice [25, 26], indicating the important role of *MTOR* in senescence, cancer, and aging. *MORF4L1* is a member of the MORF/MRG protein family that plays vital roles in cell proliferation and cellular senescence [27]. Transfected *MORF4* could induce senescence in complementation group B cell lines [28], while deficiency of *Morf4l1* in mouse embryonic fibroblasts showed reduced growth [29]. There was a lack of knowledge about the relationship between cellular senescence and the four genes *CRISPLD2*, *HAS1*, *MDK* as well as *OXTR*.

### Bypass senescence cells showed two distinct SASP patterns

**Figure 4:**
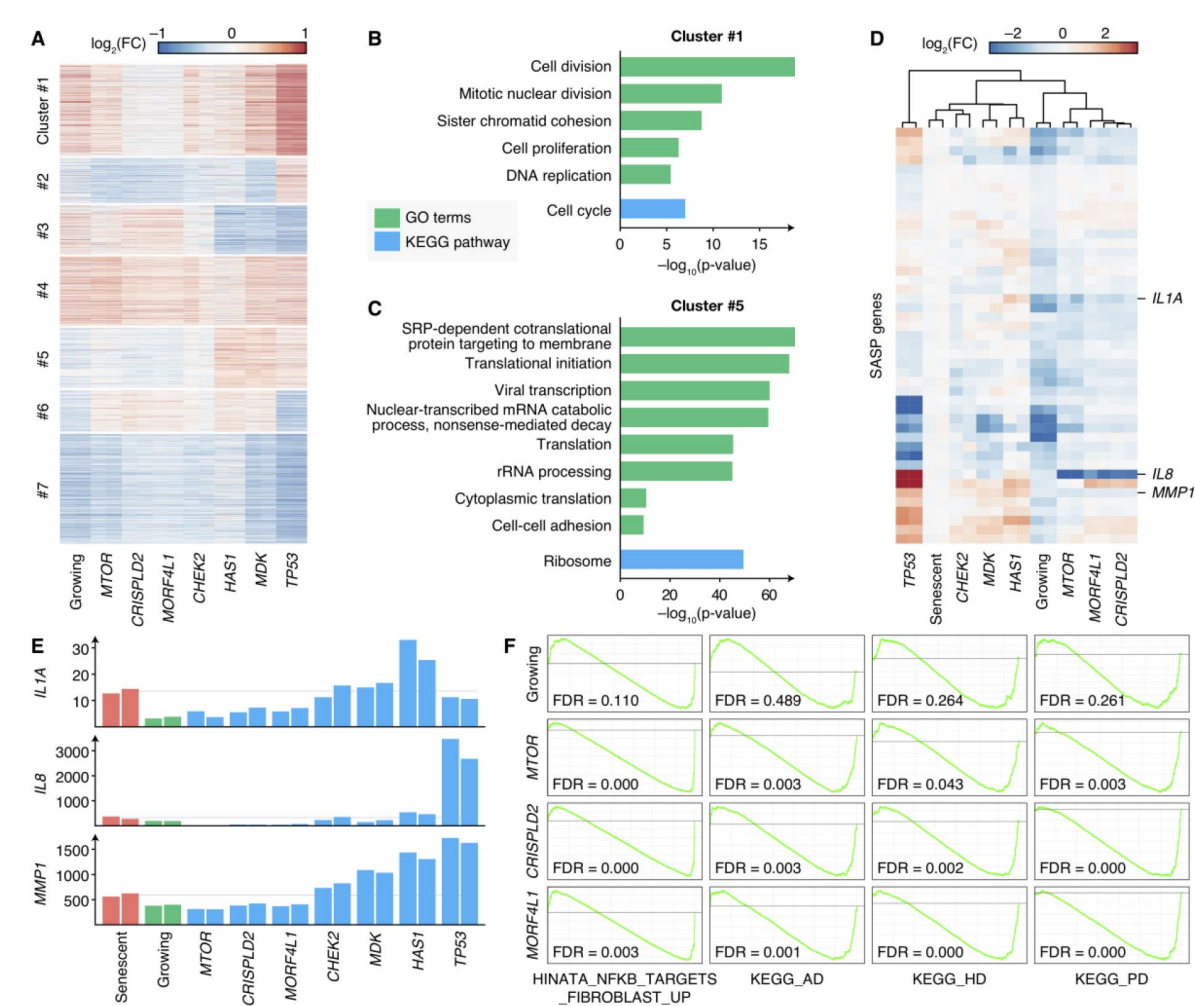
**Transcriptomes of senescence bypass cells exhibited different patterns**. (**A**) Fold changes of gene expressions of the senescence bypass cells compared with senescent cells. All differential expression genes (adjusted *p*-value < 0.05) between senescent samples and samples knocking-out validated genes were shown. Genes were clustered by k-means methods with k = 7. (**B**–**C**) KEGG pathway and GO enrichment results of genes in Cluster 1 and Cluster 5 respectively. Terms with adjusted *p*-value < 10^–5^ were shown. (**D**) Fold changes of SASP gene expression compared with senescent cells. Genes with significantly up-regulated in senescent cells compared with normal growing cells were shown (adjust *p*-value < 0.05). (**E**) Normalized expression profiles of *IL1A*, *IL8*, and *MMP1* across all RNA-seq samples. Grey lines indicated the average expression in senescent samples. (**F**) GSEA analysis of growing, *MTOR*-deficiency, *CRISPLD2*-deficiency, and *MORF4L1*-deficiency samples in the geneset up-regulated by NF-κB (HINATA_NFKB_TARGETS_FIBROBLAST_UP), KEGG pathways of Alzheimer’s disease (KEGG_AD), Huntington’s disease (KEGG_HD), and Parkinson’s disease (KEGG_PD). GSEA was performed using ranked DESeq2 Wald statistics compared with senescent cells.

Though knocking-out different genes could result in senescence bypass, the phenotypes of these cells were not the same (Supplementary Figure 3), and the mechanisms by which cells bypassed senescence were still unrevealed. Therefore, we measured the transcriptomes of bypass cells by knocking-out 6 newly-discovered senescence bypass genes (*CHEK2*, *CRISPLD2*, *HAS1*, *MDK*, *MORF4L1* and *MTOR*, excluding *OXTR* because a relatively smaller proportion of cells showed senescence bypass in *OXTR* knockout cell culture) or *TP53* respectively as well as growing cells and senescent cells by RNA-seq, each with two biological replicates. Differential expression analysis compared with senescent cells was performed, and the result showed distinct differential expression patterns (Figure 4A). The differential expressed genes were further clustered into seven gene sets by k-means clustering and then enriched by KEGG pathways and GO terms, respectively (Figure 4B, 4C and Supplementary Figure 4). A gene set (Cluster 1 in Figure 4A) in which genes were upregulated in all proliferating samples (including normal growing cells and senescence bypass cells) showed high enrichment in cell-cycle-related pathways and GO terms (Figure 4B), which was consistent with their elevated proliferative ability.

Interestingly, we found a gene set with lower expressions in *MTOR*, *CRISPLD2*, and *MORF4L1* knockout cells as well as normal growing cells compared with senescent cells, but with comparable or higher expressions in *TP53*, *MDK*, *CHEK2* and *HAS1* knockout cells (Cluster 5 in Figure 4A). This gene set was enriched in processes related to protein synthesis, such as translational initiation, rRNA processing, and protein folding (Figure 4C). Because the expression of SASP accounts for a large proportion of protein synthesis in senescent cells, we further examined the expression levels of SASP genes. 45 SASP genes identified in previous studies [30, 31] were significantly elevated in senescent cells compared with growing cells (Supplementary Table 3), and the expression of these genes differed across the senescence bypass cells (Figure 4D, 4E). Bypass cells with deficiency of *CHEK2*, *MDK* or *HAS1* showed similar SASP gene expression patterns with senescent cells, while cells with deficiency of *MTOR*, *MORF4L1* or *CRISPLD2* respectively appeared to refrain from SASP.

The results suggested two different patterns of SASP expressions in cellular senescence bypass: knocking-out *TP53*, *MDK*, *CHEK2* or *HAS1* kept the ability of proliferation when facing senescence-inducing stimuli but could not prevent cells from secreting inflammatory cytokines; while knocking-out *MTOR*, *CRISPLD2* or *MORF4L1* could keep both proliferation and non-inflammation state. A previous study has shown that the inhibition of *MTOR* could prevent SASP in senescent cells [24]. It unveiled that *MTOR* inhibition reduced IL1A translation and repressed the activity of NF-κB, which controls the expression of many SASP genes. Interestingly, we found the *IL1A* mRNA level (Figure 4E) was suppressed in cells knocking-out *MTOR*, *CRISPLD2*, or *MORF4L1* in comparison with senescent cells. What’s more, gene-set enrichment analysis (GSEA) [32] results showed a significant down-regulation within a gene set positively regulated by NF-κB [33], indicating a reduced NF-κB activity (Figure 4F). Therefore, we speculated that *CRISPLD2* and *MORF4L1* might share the similar mechanism with *MTOR* to modulate SASP through *IL1A* and NF-κB.

SASP induces inflammation, changes tissue microenvironment, and promotes age-related diseases [34]. The accumulation of senescent cells in the brain may cause chronic inflammation by SASP, affecting the pathogenesis of Alzheimer’s disease [35] and Parkinson’s disease [36]. GSEA results showed that samples with deficiency of *MTOR*, *CRISPLD2* or *MORF4L1* showed consistent down-regulation in pathways of Alzheimer’s disease, Parkinson’s disease and Huntington’s disease (Figure 4F). Moreover, *CRSIPLD2* exhibited consistent elevations in Alzheimer’s disease and Huntington’s disease patient tissues in previous studies [37] (Supplementary Figure 5). In previous studies, mTOR signaling was identified as a critical regulatory pathway in these neurodegenerative disorders [38], and inactivation of mTOR by rapamycin has been regarded to be a potential therapeutic method in the treatment of Alzheimer’s disease [39]. Correspondingly, similar transcriptome patterns of bypass samples suggested that *CRISPLD2* and *MORF4L1* may also be potential targets to deal with these diseases.

## DISCUSSION

The function of cellular senescence appears to be a paradox [2]. Senescence prevents cells from tumorigenesis, yet accumulations of senescent cells may stimulate the progression of inflammation, age-related diseases, and cancer. Therefore, understanding gene function and regulation in cellular senescence could lead to better clinical treatments of cancer or aging diseases.

Here, we carried out a CRISPR-Cas9 pooled screen and successfully discovered several novel gene deficiencies connecting to cellular senescence bypass on BJ cells. Bypassing senescence of fibroblasts usually requires inhibitions of both p53–p21 and p16–pRB pathway [5, 6]. BJ cells exhibit a low expression level of p16 in comparison with other fibroblasts such as IMR90 and WI-38, and thus are suitable for the single-gene perturbation screen. Though knocking-out genes identified from the BJ-cell-based screen may not lead to senescence bypass in other cell lines or tissues, these genes have been proved to be essential genes in cellular senescence according to the results mentioned above.

The pooled screen of senescence-related phenotypes is much more difficult compared with screens by cell viability. Thus, we put forward a novel senescence-associated sgRNA library to enhancing the screen performance. Besides, the CRISPR-Cas9 screen may trigger the DNA damage response, which is often coupled with the process of cellular senescence. Therefore, we used more stringent sgRNAs targeting non-senescence-associated genes, instead of non-target sgRNAs, as negative controls. The candidate bypass genes identified by one method usually cannot be fully recovered by another method, suggesting that these methods are non-redundant, together depicting a comprehensive landscape of senescence-associated genes. These screen results contained many known senescence bypass genes but not all, indicating the heterogeneity of senescence regulation pathways among different cell lines, induction methods, and genetic perturbation techniques [16, 40].

The bypass-senescence cells exhibit distinct transcriptome patterns. Interestingly, we found that SASP genes and pathways of some age-related neurodegenerative disorders were downregulated in senescence bypass cells induced by *MTOR*, *CRISPLD2*, or *MORF4L1* knockout. SASP plays a pivotal role to initiate and reinforce senescence and matters extremely in the paradox of cellular senescence functions [41]. Some senotherapy methodologies for cancer and aging already focused on the neutralization of SASP [42]. New genes affecting SASP as well as our screening method may provide new schemes to manipulate SASP.

There are several limitations of this study. Knocking out genes by CRISPR-Cas9 cannot guarantee complete deficiencies of target genes on both alleles, elevating the noise in pooled screen and validation. Besides, the populations of senescent cells are highly heterogeneous [40, 43], and the method cannot distinguish whether only a subpopulation of cells bypass senescence. Moreover, the pooled screen method cannot discriminate senescence bypass and senescence delay, and the dynamics of cellular senescence bypass should be characterized by time-series data. Finally, the regulation of senescence SASP is highly orchestrated and context-dependent *in vivo*, thus the mechanisms of how deficiencies of *CRISPLD2* and *MORF4L1* cause senescence bypass and SASP reduction are far from clear. Previous studies reported that MRC5 cells with *CRISPLD2* knockdown showed exacerbated expressions of some SASP genes such as IL-6 and IL-8 when inducing inflammation by lipopolysaccharide [44], which may seem to be conflicted with our results. Therefore, the regulation between *CRISPLD2* and SASP genes may depend on the cell type and the extracellular stimuli.

Our findings suggested that senescence bypass could be driven by different mechanisms and could lead to distinct consequences. Senescent cells with the disruption of some genes may escape from senescence but cause inflammation, thus increasing the risk to tumorigenesis [45, 46]. However, manipulating some other genes may have the ability to remain proliferation while refraining from the secretory phenotype, which would potentially slow down the aging process. Although we have only presented the initial evidence on the hypothesis, these results have painted a remarkable picture of how delicate it is if trying to bypass senescence to keep vigor without causing cancer or aging.

## MATERIALS AND METHODS

### Cell culture

Human foreskin fibroblasts BJ cells were obtained from ATCC (Manassas, VA, USA) and cultured in Dulbecco’s modified Eagle’s medium (DMEM, Life Technologies, Carlsbad, CA, USA) with 10% fetal bovine serum (FBS, Life Technologies) and 1% penicillin/streptomycin (Life Technologies) at 37°C in a 5% CO_2_ incubator, and passaged at a ratio 1:3 every 3–4 days. BJ-Cas9 cells that stably expressed Cas9 were authenticated by STR multi-amplification (HKgene, Beijing, China). HEK293T cells were maintained in DMEM with 10% FBS and 1% penicillin/streptomycin. Cells were passaged at a ratio of 1:5 every two days.

### Vectors

Human Cas9 lentiviral plasmid was obtained from Prof. Lei S. Qi’s lab (Stanford University, Stanford, CA, USA). The lentiviral sgRNA vector was obtained from SyngenTech (Beijing, China).

### sgRNA library design

Genes related to cellular senescence were designed by integrating the information of literature mining, PPI network, and senescence differential expression datasets. Firstly, literature mining was conducted to identify known cellular senescence genes through the method LMMA [47]. With the query “cellular senescence”, 1507 genes were identified from the collected 3597 articles. 620 genes that occurred in more than three articles or occurred in the articles with a sum of impact factors (IFs) larger than nine were left as candidate genes. Besides, 89 genes were collected manually from gene sets associated with DNA damage response dependent and independent pathway, SASP, and insulin growth factor pathway. Secondly, 330 genes were collected from the PPI network in HCSGD (version in December 2014) [17]. Thirdly, senescence-associated genes were identified from the differential expression analysis of collected 15 series of microarray data from GEO database through a rank-based meta-analysis method RankProd [48]. A DAVID GO enrichment [49] was performed on the genes with FDR < 0.01. The final gene set (659 genes) was composed of the genes in the enriched GOs. In total, 1391 genes were chosen as potential senescence-associated genes.

Negative control genes were chosen by some filtering rules. The candidate genes were genes occurring in all three different microarray platforms (133plus2, Exon1.0 ST and Human Gene 1.0 ST) used in the meta-analysis. Genes in the literature mining results, in the PPI network as well as in the manually-chosen list were filtered out. Then genes with adjusted *p*-values less than 0.5 in the meta-analysis result or with average fold change more than 1.3 were filtered out (203 genes). Genes occurring in the literature-mining result by LMMA with the keyword “senescence” or “apoptosis” or “cell death” or “proliferation” or “cell cycle” or “autophage” or “quiescence” were filtered out. Only 63 genes were left.

We created a sgRNA library targeting 1378 genes associated with cellular senescence and 56 negative control genes. Each gene was targeted by eight sgRNAs on average, designed by CRISPR-ERA [18]. The whole library contained about 12000 sgRNAs.

### CRISPR-Cas9 screening

Virus packaging HEK293T cells were plated onto 100-mm cell culture dishes (3×10^6^ cells per dish) 24h before transfection. For each dish, 1 μg of plasmids containing transducing vector, 2 μg of pCMV-dR8.2 dvpr (Addgene, 8455) and 1 μg of pCMV-VSV-G (Addgene, 8454) were transfected using 20 μl Attractene Transfection Reagent (Qiagen, Germantown, MD, USA). Virus supernatant was harvested 48h and 72h after transfection and then filtered with a 0.45-μm sterile Acrodisc Syringe Filter with Supor Membrane (Pall Corporation, Port Washington, NY, USA). The virus carrying the sgRNA library was concentrated using lenti-concentin virus precipitation solution (Genomeditech, Shanghai, China), aliquoted, and stored at –80 °C.

3×10^7^ BJ-cas9 cells were plated onto 150-mm dishes with 2.2×10^6^ cells per plate. 24 hours later, cells were infected with sgRNA library virus at an MOI of 0.22 (>500 cells per sgRNA) and selected with 2 μg/ml puromycin for five days. Cells were maintained at more than 550 cells per sgRNA during subsequent culture.

On day 12 after infection, 6.6×10^6^ BJ-cas9-sgRNAlib cells were harvested as control 0d. The other cells were induced to senescence by 40 μg/ml bleomycin sulfate (Biorbyt, Cambridge, UK) for two hours for the bleomycin-induced screen, or infected with H-Ras^V12^ lentivirus for the Ras-induced screen. Each condition contained 6.6×10^6^ cells (550 cells per sgRNA). These cells were passaged after two or three days. Ras-induced cells were harvested on day 13 after induction, while bleomycin-induced cells were harvested on day 27 after induction, each with over 500 cells per sgRNA for genomic DNA extraction. Two independent replicates of the library screen were performed from the beginning of sgRNA library virus transduction for the bleomycin-induced screen.

### sgRNA sequencing and analysis

Genomic DNA was isolated with DNeasy Blood and Tissue kit (Qiagen). To identify sgRNAs, we amplified the DNA fragments flanking sgRNA-coding region by PCR using a high-fidelity DNA polymerase (NEB, Ipswich, MA, USA) with two primers (Supplementary Table 2). The amount of input DNA was 64 μg for each replicate (>200 coverage over CRISPR-sgRNA library). For details, 1 μg DNA template was used in 50 μl PCR reaction. 64 parallel PCR reactions were carried out for each replicate, and the PCR products of 64 tubes were pooled and purified with DNA Clean & Concentrator-5 (Zymo Research, ‎Irvine, CA, USA), following by next-generation sequencing using Hiseq 4000.

As the sequence of sgRNA PCR product is much shorter than DNA sequencing read length, raw pair-end sequencing reads were merged into long reads using FLASh v 1.2.11 [50]. Sequences between two consistent flanking sequences (Supplementary Table 2) with a certain length (19bp) were extracted and aligned to the sgRNA sequences of the plasmid library with no mismatch allowed.

### Scoring and identifying candidate genes by MA-plot methods

To identify sgRNAs which levels are distinct between case and control samples, we used an MA-plot-based method to score each sgRNA. First, we filtered out control sgRNAs which levels were in the top 10% or the bottom 10% in any sample and then calculated the median of the rest control sgRNAs’ reads count as control-median. The reads count of all sgRNAs in each sample was then normalized using the control-median of this sample.

For any senescence-induced sample, let *C_i_*^+^ denote the normalized reads count of sgRNA *i* in this sample and *C_i_*^–^ denote the normalized reads count of sgRNA *i* in the corresponding control sample. Then we defined *A_i_* = (lg *C_i_*^+^ + lg *C_i_*^–^)/2 and *M_i_* = lg *C_i_*^+^ – lg *C_i_*^–^. A sliding window (each window contains 2% of the control sgRNAs) was applied on the *A*-axis, and the standard deviation of *M* (*σ_M_*_|*A*_) in each window was obtained. We fitted an exponential model between *σ_M_*_|*A*_ and *A* using the R package *nls*: *σ_M_*_|*A*_ ~ *αe*^–*βA*^. Finally, for any sgRNA *i*, we obtained a Z-score under this estimated deviation: $Z_{i}=M_{i}/{\hat{\sigma }}_{M|A_{i}}$. The *p*-value of each sgRNA was calculated from its Z-score. We identified a gene as a candidate gene if there are 3 or more sgRNAs of this gene significant (*p*-value < 0.05) in both replicates.

### Validation of individual sgRNAs

2.2×10^5^ BJ-Cas9 cells were plated onto 60 mm dishes and infected with lentivirus supernatant of individual sgRNAs. At day 3 post-infection, cells were selected with 2 μg/ml puromycin (Life Technologies) for five days, following with passage. On day 9 after infection, cells were induced to senescence by 40 μg/ml bleomycin for 2 hours. 2-3 days later, all of the cells were passaged. On day 24 after treating with bleomycin, cells were plated in 12-well plates, 6-well plates and confocal dishes respectively to perform the senescence-associated β-gal assay, colony formation and proliferative marker Ki67 immunofluorescence assay.

### Senescence-associated β-gal assay

Cells were plated in 12-well plates at a density of 6×10^4^ cells/ml. Three days later, cells were stained with a β-gal assay kit (BioVision, Milpitas, CA, USA) [51], and at least 100 cells were analyzed for each well.

### Colony formation assay

Cells were plated in 6-well plates with 5000 cells per well, and cultured for 10-14 days. Cells were fixed with methanol for 15 min, washed slowly with running water, stained with Giemsa working solution for 30 min, and then washed again.

### Immunofluorescence assay

Cells were plated onto 35mm confocal dishes and cultured for at least three days. Cells were washed with phosphate buffer saline (PBS) three times and fixed with 4% paraformaldehyde. After blocking with 5% bovine serum album (BSA), cells were incubated with anti-Ki67 (ab16667, Abcam) at 4°C overnight, washed with washing buffer (PBS, 0.2% Tween-20), incubated with anti-rabbit Alexa Fluor 488 secondary antibody (ab150073, Abcam) at 1:400 for 1 hour at room temperature, and then washed three times and finally stain nuclear with DAPI. Cells were observed with a laser scanning confocal microscope and analyzed at least 100 cells per dish.

### RNA-seq

Total RNA was isolated from cells and purified using poly-dT oligo-attached magnetic beads. Fragmentation was generated, first strand cDNAs were synthesized with random hexamer primer, and second strand cDNAs were synthesized using DNA Polymerase I and RNase H. The cDNAs were ligated with adaptors and produced libraries by NEBNext Ultra RNA Library Prep Kit for Illumina (NEB). Libraries were sequenced on Illumina HiSeq 4000.

### RNA-seq analysis

The RNA-seq reads were mapped to hg19 using TopHat v 2.1.1 [52]. Gene expression reads were counted by HTSeq v 0.6.1 [53]. Differential expression was performed by DESeq2 [54]. The gene expression profiles were normalized by sequencing depth. All genes that were significantly differentially expressed (adjusted *p*-value < 0.05) between senescence cells and samples with any newly-discovered senescence bypass gene knockout were carried out for k-means (k = 7) clustering by their logarithmic fold changes.

### Gene enrichment and gene network analysis

GO and KEGG enrichments were performed by DAVID v 6.8 [49]. The *p*-value was adjusted using the Benjamini-Hochberg procedure. Gene-set enrichment analysis for RNA-seq data was performed using ranked DESeq2 Wald statistics by GSEA v 3.0 [32]. The PPI network was generated by the STRING database [55].

### Time-series microarray data analysis

Time-series microarray data GSE41714 were divided into four groups (Group 1: Day 2; Group 2: Day 3–7; Group 3: Day 10–20; Group 4: Day 30 and after) according to the related research [21]. Differential expression was then analyzed by GEO2R. Genes with FDR < 0.05 were identified to be differentially expressed.

### AD and PD patient data analysis

Three datasets of neurodegenerative patient tissues, including GSE95587 (AD, fusiform gyrus) [37], GSE15222 (AD, temporal cortex) [56], and GSE3790 (HD, caudate nucleus) [57] were analyzed. The data and analysis results were obtained from The Myeloid Landscape (http://research-pub.gene.com/BrainMyeloidLandscape) [37].

## Supplementary Material

Supplementary Figures

Supplementary Tables
