# Supplementary material for: A large-scale CRISPR screen and identification of essential genes in cellular senescence bypass
Source: Aging (Albany NY). 2019 Jun 20;11(12):4011–31. doi: 10.18632/aging.102034 (PMC6628988; doi:10.18632/aging.102034)
Supplement: Supplementary Figures [file aging-11-102034-s002.pdf]

SUPPLEMENTARY FIGURES

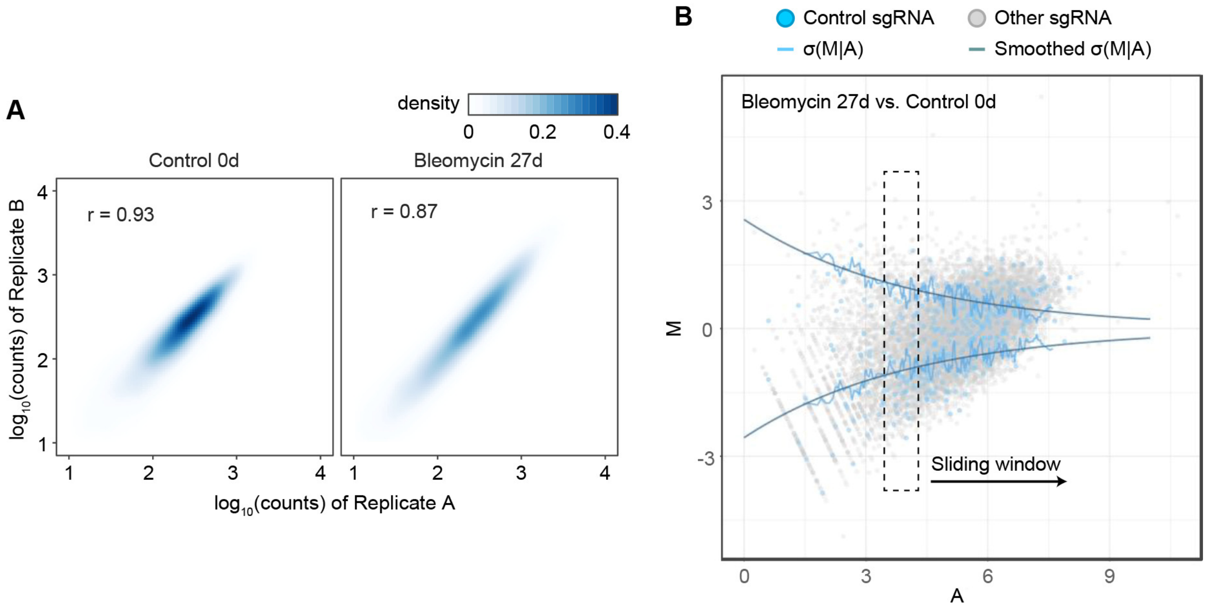

Supplementary Figure 1. The large-scale CRISPR knockout screen and analysis. (**A**) Replicates of CRISPR screen samples showed high correlation. Pearson correlations (r) were given. (**B**) M-A plot methods for scoring and identifying candidate senescence bypass genes.

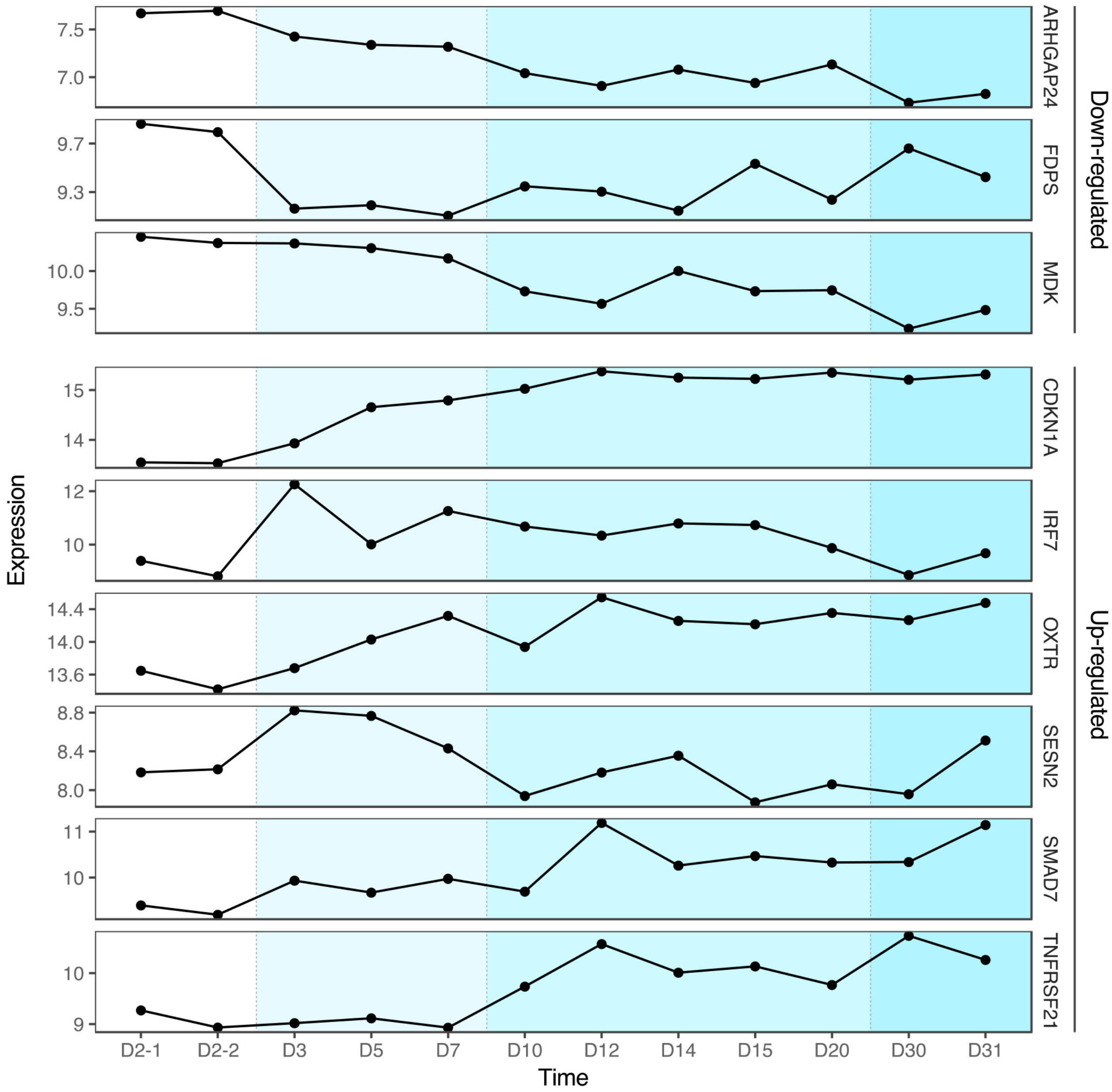

Supplementary Figure 2. Expressions of the candidate bypass genes during senescence. The time-series gene expression data are from dataset GSE41714 (replicative senescence, human diploid fibroblasts). Colors represent groups for differential analysis. Only genes with differential expression (adjust *p*-value < 0.05) were shown.

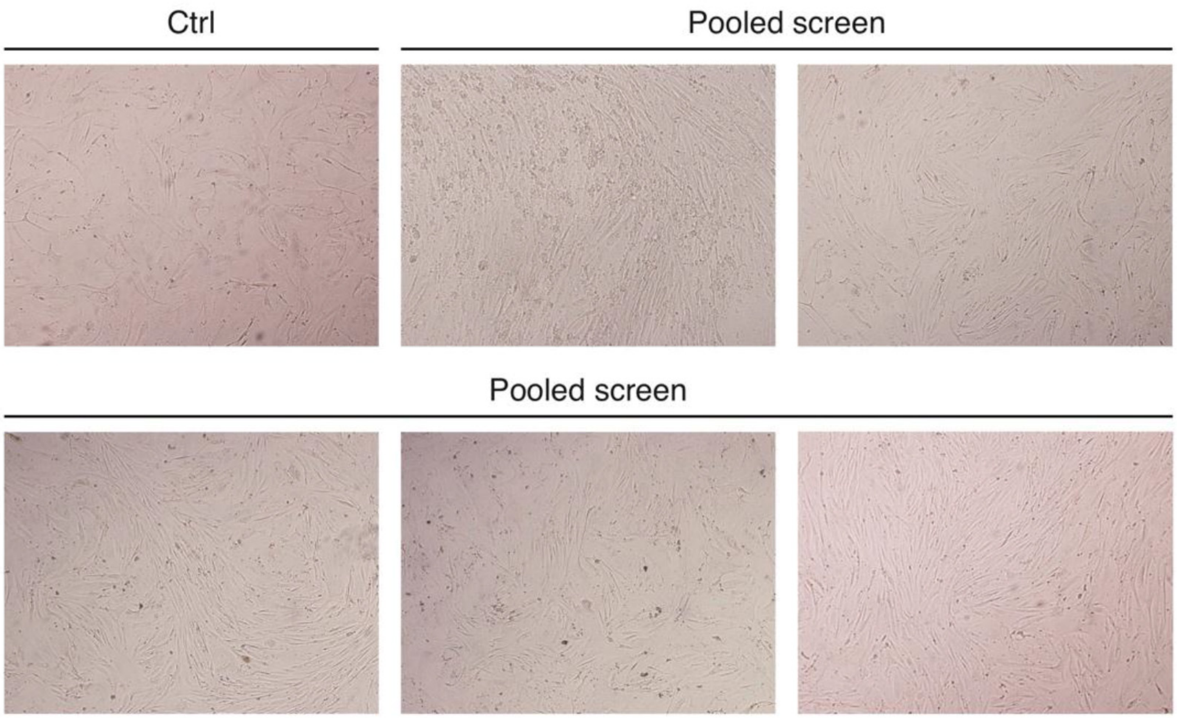

Supplementary Figure 3. Representative images of various colony shapes in the pooled screen.

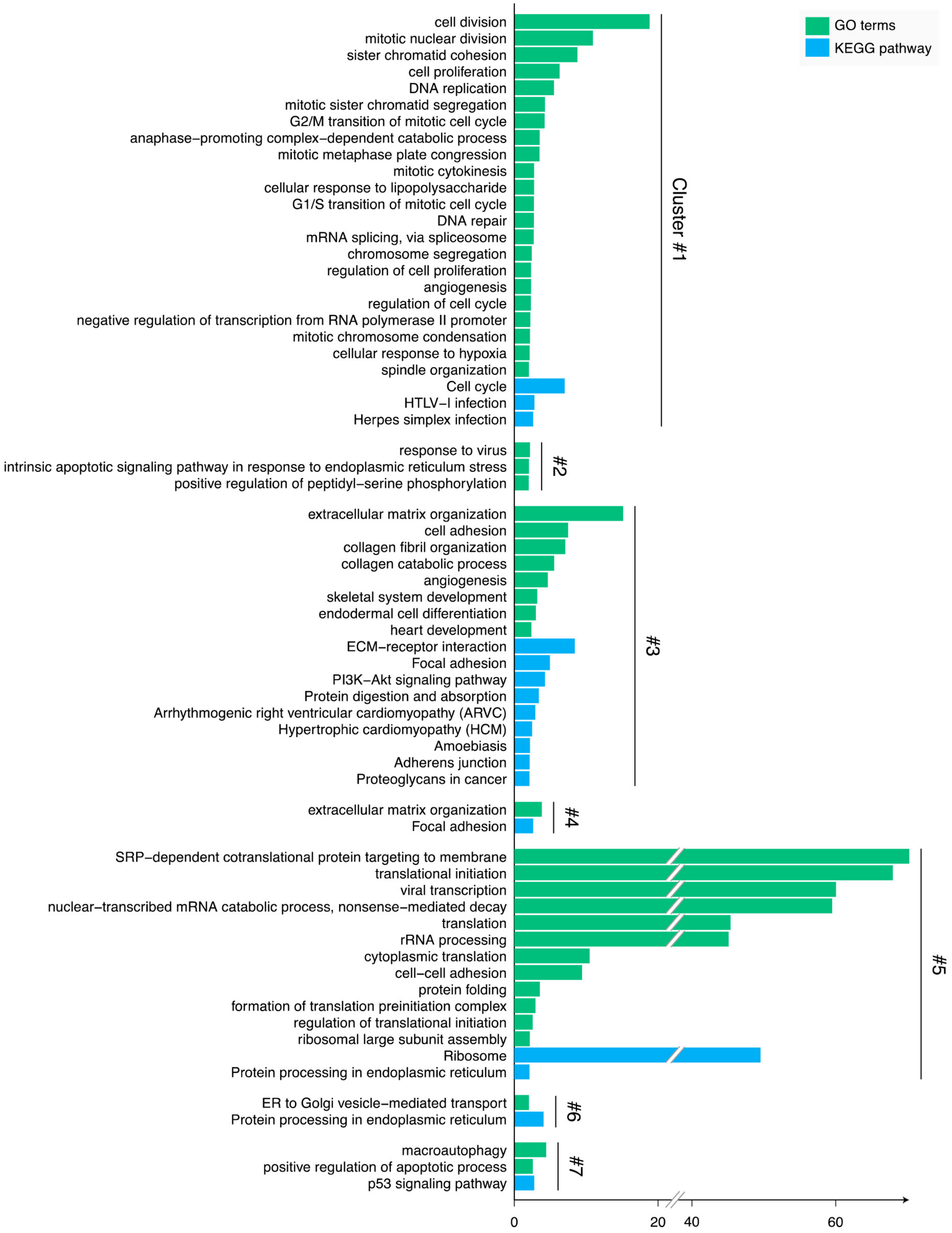

Supplementary Figure 4. KEGG pathway and GO enrichment results of genes in each cluster in transcriptome data. Terms with adjusted *p*-value < 0.01 were shown.

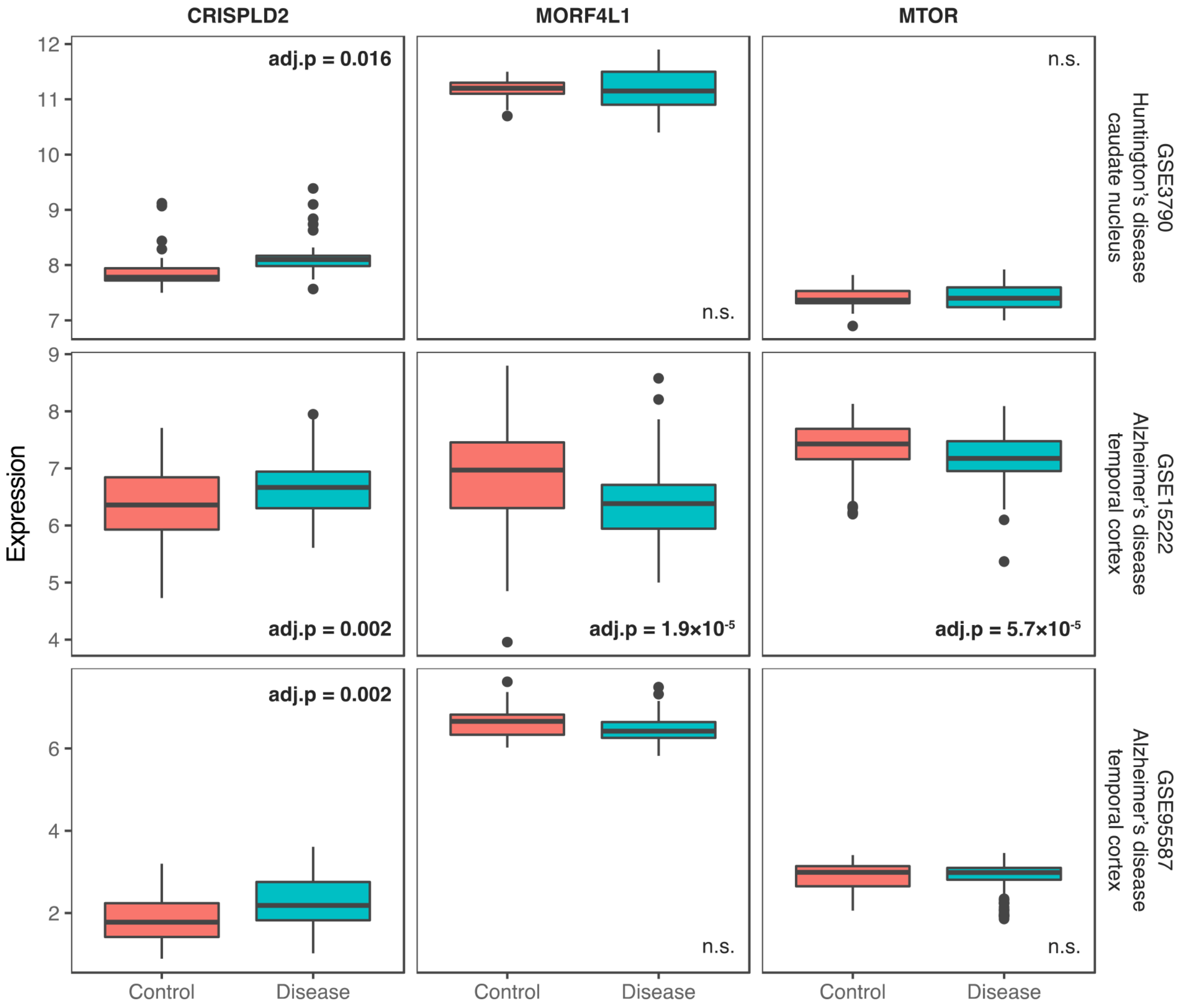

Supplementary Figure 5. Expressions of *CRISPLD2*, *MORF4L1*, and *MTOR* in Alzheimer's disease and Huntington's disease patient tissues in previous studies. Data and statistics were obtained from The Myeloid Landscape (http://research-pub.gene.com/BrainMyeloidLandscape).
