# Supplementary material for: A large-scale CRISPR screen and identification of essential genes in cellular senescence bypass
Source: Aging (Albany NY). 2019 Jun 20;11(12):4011–31. doi: 10.18632/aging.102034 (PMC6628988; doi:10.18632/aging.102034)
Supplement: Supplementary Tables [file aging-11-102034-s001.pdf]

## SUPPLEMENTARY TABLES

**Supplementary Table 1. Candidate bypass genes identified by CRISPR-based screen.**

| Gene name | Sources to select senescence-associated genes from | | | | Previously reported bypass genes | Validation | |
|---|---|---|---|---|---|---|---|
| | Literature mining | Differential expression | PPI networks | Collecting manually | | Further validation | Successfully validated |
| AHNAK2 | | 1 | | | | 1 | |
| AKT1 | 1 | | | | [1] | | |
| ARHGAP24 | 1 | | | | | 1 | |
| ARID3A | | | 1 | | [2] | | |
| ATM | 1 | | 1 | 1 | [3] | | |
| CASP9 | 1 | | 1 | | | | |
| CDKN1A | 1 | 1 | 1 | 1 | [4] | | |
| CHEK2 | 1 | 1 | 1 | 1 | | 1 | 1 |
| CRISPLD2 | | 1 | | | | 1 | 1 |
| FDPS | 1 | | | | | | |
| FOSL1 | 1 | | | | | | |
| GCLC | | 1 | | | | | |
| GPSM1 | | | | | | 1 | |
| HAS1 | | 1 | | | | 1 | 1 |
| IKBKB | 1 | | | | | 1 | |
| IRF7 | 1 | | | | | | |
| ITSN2 | 1 | | | | [5] | | |
| MDK | | 1 | | | | 1 | 1 |
| MIF | 1 | | | | | | |
| MORF4L1 | 1 | | 1 | | | 1 | 1 |
| MTOR | 1 | | 1 | 1 | | 1 | 1 |
| NDUFB1 | | | | | | | |
| OGG1 | 1 | | | | | | |
| OXTR | | 1 | | | | 1 | 1 |
| POT1 | 1 | 1 | | | | 1 | |
| SESN2 | | | | 1 | | 1 | |
| SMAD7 | | 1 | 1 | | | | |
| TNFRSF21 | | 1 | | | | | |
| TP53 | 1 | | 1 | 1 | [6] | 1 | 1 |
| ZBTB7A | 1 | | | | [7] | | |

1. Nogueira V, Park Y, Chen CC, Xu PZ, Chen ML, Tonic I, Unterman T, Hay N. Akt Determines Replicative Senescence and Oxidative or Oncogenic Premature Senescence and Sensitizes Cells to Oxidative Apoptosis. Cancer Cell. 2008; 14:458–70. https://doi.org/10.1016/j.ccr.2008.11.003 PMID:19061837

2. Peeper DS, Shvarts A, Brummelkamp T, Douma S, Koh EY, Daley GQ, Bernards R.. A functional screen identifies hDRIL1 as an oncogene that rescues RAS-induced senescence. Nature Cell Biology. Nat Cell Biol. 2002; 4:148–53. https://doi.org/10.1038/ncb742 PMID:11812999

3. Aird KM, Worth AJ, Snyder NW, Lee JV, Sivanand S, Liu Q, Blair IA, Wellen KE, Zhang R. ATM Couples Replication Stress and Metabolic Reprogramming during Cellular Senescence. Cell Rep. 2015; 11:893–901. https://doi.org/10.1016/j.celrep.2015.04.014 PMID:25937285

4. Brown JP, Wei W, Sedivy JM. Bypass of Senescence After Disruption of p21CIP1/WAF1 Gene in Normal Diploid Human Fibroblasts. Science. 1997; 277:831–4. https://doi.org/10.1126/science.277.5327.831 PMID:9242615

5. Uma Karthika Rajarajacholan, Subhash Thalappilly, Karl Riabowol. The ING1a Tumor Suppressor Regulates Endocytosis to Induce Cellular Senescence Via the Rb-E2F Pathway. PLoS Biol. 2013; 11:e1001502. https://doi.org/10.1371/journal.pbio.1001502 PMID:23472054

6. Sherr CJ, McCormick F. The RB and p53 pathways in cancer. Cancer Cell. 2002; 2:103–12. https://doi.org/10.1016/S1535-6108(02)00102-2 PMID:12204530

7. Wang G, Lunardi A, Zhang J, Chen Z, Ala U, Webster KA, Tay Y, Gonzalez-Billalabeitia E, Egia A, Shaffer DR, Carver B, Liu XS, Taulli R, et al. Zbtb7a suppresses prostate cancer through repression of a Sox9-dependent pathway for cellular senescence bypass and tumor invasion. Nat Genet. 2013; 45:739–746 https://doi.org/10.1038/ng.2654 PMID:23727861

**Supplementary Table 2. Primers and validation sgRNAs.**

| | | |
|---|---|---|
| **Primers for sgRNA sequencing** | ACTTGCTATTTCTAGCTC | TCATATGCTTACCGTAACTTG |
| **Flanking sequences for sgRNA identification** | GTGGAAAGGACGAAACACCG | GTTTTAGAGCTAGAAATAGC |
| **sgRNAs for validation** | | |
| Gene | sgRNA1 | sgRNA2 |
| AHNAK2 | TTTCTGCACCTGGCTCCCC | CTCCCCGGGCTGCAGCTGA |
| ARHGAP24 | AACCGTCTGGCTCCGATGT | CCGCCCTTGGCCTTGTTGG |
| CHEK2 | GATATGCCCTGGGACTGTG | GACTGGGTAACGCTGCCAT |
| CRISPLD2 | GGAGTGAGACTCGTTGTGC | CACAACAAGCTTCGGGGCC |
| GPSM1 | AATTCCTCGGTCTGGGGCG | AGGTGCCCTGCATTGCTGT |
| HAS1 | CTGCGCGTCCTCATGGTGG | CCTTCGCCCTGCTCATCCT |
| IKBKB | CCCAATGACCTGCCCCTGC | GCACCACCGCTCTCGGTTC |
| MDK | CGCTACAATGCTCAGTGCC | CACCGGATGCGCTGGGTCT |
| MORF4L1 | GAGGGTGAGTGTGCGCCTT | GCGGTCAGTGCTTTGTGCG |
| MTOR | ACGGAAACCGTAGCTGCCC | GCAGCTACGGTTTCCGTCT |
| OXTR | ACCGTGCGGATCTTGGCCT | ACTCCACGGACGGATCTGC |
| POT1 | TTCACACACTGATGGCGCC | TTACGCAGCTGGTCCACCG |
| SESN2 | ACAACCTGGCAGTGGTGAT | ACCATCCGTGTGCAGCAGC |
| TP53 | GCAGCTACGGTTTCCGTCT | ACGGAAACCGTAGCTGCCC |

**Supplementary Table 3. List of SASP genes for analysis in Figure 4D.**

| | | | | |
|---|---|---|---|---|
| AREG | GAL | LIF | NTM | SDF4 |
| BMP2 | GDF15 | LTBP1 | NTNG1 | SERPINB2 |
| C3 | GDNF | MMP1 | PI3 | SFRP1 |
| CCL26 | IGF1 | MMP10 | PLAT | SIL1 |
| CDCP1 | IGFBP5 | MMP3 | PLAU | SRPX2 |
| CHID1 | IL1A | NAMPT | PTX3 | STC1 |
| EGF | IL8 | NPTX1 | PVR | TFRC |
| ESM1 | KITLG | NRG1 | QPCT | TNFRSF10C |
| FGF2 | LAMC2 | NT5E | SCG2 | TNFRSF10D |
